# Carbohydrate confusion and dietary patterns: unintended public health consequences of “food swapping”

**DOI:** 10.3389/fnut.2023.1266308

**Published:** 2023-09-28

**Authors:** Keith T. Ayoob

**Affiliations:** Department of Pediatrics, Albert Einstein College of Medicine, Montefiore Hospital and Medical Center, Bronx, NY, United States

**Keywords:** potatoes, nutrition, starchy vegetables, carbohydrate quality, diet quality, nutrients of concern, dietary guidelines

## Abstract

The 2025–2030 United States Dietary Guidelines process is currently underway, and the 2025 Dietary Guidelines Advisory Committee is examining and evaluating a list of prioritized scientific questions identified by the United States Department of Health and Human Services and the United States Department of Agriculture. One of the questions that will be evaluated is if changes should be made to USDA Dietary Patterns based on whether starchy vegetables and grains are, or can be, consumed interchangeably. These foods have historically been classified in distinct food groups. Menu modeling analyses evaluating the impact of replacing starchy vegetables with grains result in declines in key nutrients of concern. Given their unique nutrient contributions and the fact that many cultural foodways within the United States population include both starchy vegetables and grains, it is important for dietary recommendations to continue to categorize starchy vegetables and grains separately.

## Introduction

Foods rich in carbohydrates, such as grains, fruits, legumes, nuts and seeds, and vegetables, including starchy vegetables, provide the bulk of dietary intake for most global cuisines and food patterns (1). Grain crops alone, namely maize, wheat, and rice, comprise 51% of the total kilocalories consumed globally, while roots and tuber crops comprise 5.3% (2).

As the work of the 2025–2030 U.S. Dietary Guidelines Advisory Committee (DGAC) continues, the U.S. Department of Health and Human Services (HHS) and U.S. Department of Agriculture (USDA) released a list of proposed scientific questions that will inform the work of the DGAC (3). Among key questions being evaluated include:

- Considering each life stage, should changes be made to the USDA Dietary Patterns (Healthy United States-Style, Healthy Mediterranean-Style, and/or Healthy Vegetarian), and should additional Dietary Patterns be developed/proposed based on:
  - Findings from systematic reviews, data analysis, and/or food pattern modeling analyses.
  - Population norms (e.g., are starchy vegetables often consumed interchangeably with grains?), preferences (e.g., emphasis on one staple grain vs. another), or needs (e.g., lactose intolerance) of the diverse individuals and cultural foodways within the United States population?

Notably, the DGAC is considering if starchy vegetables can be considered as interchangeable with grains. This perspective substantiates the need to consider the diversity of complex carbohydrate-containing foods, each with a unique role to contribute to overall nutrition and dietary health. Nutrient-dense dietary patterns include both grain foods and starchy vegetables. These food groups are currently considered separately, and they must remain separate to ensure people are encouraged to consume complementary nutrients from each of these food groups.

Further, this paper includes a menu modeling analysis which demonstrates the nutritional differences between starchy vegetables and grains in an overall dietary pattern. Using complex carbohydrate foods, specifically starchy vegetables (e.g., potatoes) and grains, interchangeably is at best, not a useful strategy, but at worst, may increase the risk of micronutrient inadequacy and/or dietary imbalances.

## Carbohydrate foods: a spectrum of composition and consumer use

### Complex carbohydrate foods have varied forms and structures

Carbohydrate foods are often lumped into one macronutrient category, despite their wide nutritional variations. Simple carbohydrates, such as mono-and disaccharides, can be intrinsically present in foods, as in fruit, or isolated and used as an ingredient, as for sucrose from cane sugar.

“Complex” carbohydrates can be even more varied. Starches and fibers can have different lengths and compositions. Even fiber, a subclass of complex carbohydrates, is far from homogenous, with its various soluble, insoluble, cellulose, hemicellulose, pectins, lignins, and variants (4). Dietary fiber is found only in foods already rich in carbohydrates, unless specifically added through processing.

Structurally, carbohydrates have a common glycerol backbone, but otherwise their chemical structures are broad, divided into various mono-, di-, and polysaccharides, resulting in differing degrees of digestibility, bioavailability, and function.

The term “starch” can also have multiple meanings. Most starch is easily broken down into its component simple carbohydrates, but “resistant starch” (RS) passes to the large intestine intact and undigested. Even RS is known to have five variants (5). Two such variants are relevant to this discussion:

- RS3: also known as retrograded starch, forms after cooking high-starch foods like potatoes or rice, then cooling and refrigerating them, causing their long amylose chains to recrystallize into double helices that amylase cannot break down.
- RS5: formed when starchy foods are fried, producing a starch-lipid matrix.

Resistant starch has been widely studied in recent years, with studies showing possible benefits to lipid metabolism, body weight, and as a prebiotic to improve gut health (5). Resistant starch has also been demonstrated to be particularly effective at promoting a healthy microbiome (6). This emerging evidence suggests that the role of “starch,” refined or whole, may be more varied, and more valuable, than merely as a component of complex carbohydrates.

### Culinary use of carbohydrate foods is widely varied

Some carbohydrate foods, such as fruits and vegetables, can be eaten alone, even raw, while others are more commonly used as ingredients, either in cooked meals or snacks, or as ingredients, such as grain flour in breads and baked goods. Carbohydrate-containing foods can be eaten in savory or sweet preparations, allowing them a versatile role in the diet.

Wheat, rice, and maize (corn) are the three most commonly consumed grains in the world (7). Even in refined and enriched form, that is, containing mostly the starchy endosperm, without the germ or outer bran layer, these staple grains often serve as “vehicle foods” for delivering vegetable and protein components of meals. Starchy grains have long been a culinary staple of many cultures, but also as indulgent foods for lower-nutrient savory or sweet snacks and desserts. Starchy vegetables, such as potatoes, are vegetables in their own right and also serve as “vehicles” or delivery mechanisms for additional nutrient-dense foods, such as other vegetables, healthy fats, and lean proteins.

In many cultures, starchy vegetables and grains have also provided an economical means of “stretching” modest amounts of proteins while still providing a satiating and nutrient-dense meal. Examples include combining potatoes with eggs in a Spanish frittata (Tortilla de Patatas) or a skillet meal that mixes lean ground beef with potatoes and vegetables. These two dishes have their variations in many cuisines and provide a way to extend more moderate amounts of proteins, while increasing overall vegetable consumption.

Starchy vegetables, in contrast to grains, are seldom consumed with added sugar, either as a staple vegetable in a meal, or as a snack, the exception being candied sweet potatoes or sweet potato pie, but overall consumption of these is low. The term “grain-based desserts,” for instance, does not have a potato equivalent. Whether starchy vegetable or starchy grain, either can contain added fats and/or sugars. Dietary balance, which includes consideration for quantity and frequency of consumption, remains a cornerstone of dietary guidelines.

## Starchy vegetables and grains have significantly different nutrition profiles

Grains and starchy vegetables share the commonality of being complex carbohydrates, and in the case of whole grains, fiber as well. However, that is where much of their similarities end.

Significant differences between grains and starchy vegetables exist in their micronutrient content. Starchy vegetables, such as potatoes, are more known for their starch content than for their potassium, iron, B_6_, and vitamin C content. Potatoes are a good source of potassium, one of the “nutrients of public health concern” identified by the 2020–2025 United States Dietary Guidelines for Americans (8) (DGA, 2020–2025). A medium-sized potato with skin (5.3 oz) and two slices of whole wheat bread (two slices) have similar energy value, approximately 110 vs. 160 kcal, respectively. However, the following are among their differences in nutrient content (9, 10):

- Potassium:
  - Medium-sized potato: 15% daily value (DV)
  - Whole wheat bread: 3% DV
- Vitamin C:
  - Medium-sized potato: 30% DV
  - Whole wheat bread: 0%
- Vitamin B6:
  - Medium-sized potato: 10% DV
  - Whole wheat bread: 8% DV
- Iron:
  - Medium-sized potato: 6% of the DV
    - Notably, the low phosphorous content of potatoes makes their iron more bioavailable than the iron in plant foods with higher phytate content (11). Their higher vitamin C content also aids iron absorption.
  - Whole wheat bread: 9% DV
- Fiber:
  - Medium-sized potato: 8% of the DV
  - Whole wheat bread: 14% of the DV

Grains and starchy vegetables both lose fiber when processed by removal of the bran and germ from grains, and removal of the skin from potatoes. If potatoes are boiled, there can be additional loss of potassium through leeching. Mineral content is well retained whether potatoes are baked, fried, roasted, or microwaved (12). Frying and baking, because they decrease moisture content, actually increase the concentration of key nutrients, including potassium.

Compared with starchy vegetables, such as potatoes, grains tend to be lower in potassium and vitamin C, but intrinsically provide higher amounts of other nutrients, such as thiamine, zinc, and vitamin E. If grains are consumed in “whole” form, their bran and germ components can be sources of phytochemicals, mostly as polyphenols (13). The majority of grain foods purchased however, are in the form of refined grains, where the germ and bran layers, and the nutrients they contain, are removed (14). In the United States, refined grains are required to contain added iron, folic acid, riboflavin, niacin, and thiamin, given these nutrients are lost during the refinement process (15).

Among macronutrient differences between grains and potatoes, whole wheat bread contains 7.2 g of protein in two slices (64 g), compared with 3 g in a medium-sized potato (5.3 oz) of similar energy content (9, 10). While neither food is considered a major protein source, the protein quality in potatoes is superior, with a biological value (BV) of 90 (16), which is comparable to the BV of protein in egg and milk (11, 17) and higher than the BV of other excellent plant protein sources, including soybeans (16).

## Classification of grains and starchy vegetables in scientific literature

Tools for collecting dietary intake data have varied over time, and have included various questionnaires, 24-h recalls, and interviews. The National Health and Nutrition Examination Survey (NHANES) uses trained interviews to conduct dietary recalls on two separate days (18). Other large-scale studies have used a semi-quantitative food frequency questionnaire (FFQ), often using one developed by the Harvard School of Public Health (19).

These data collection tools do not always consistently classify foods, especially starchy vegetables. The USDA and the DGAs consider all white potatoes as vegetables. Yet, the FFQ used in many large observational studies, groups white potatoes with breads, cereals, and starches, but other starchy vegetables, such as sweet potatoes, including sweet potato fries, and butternut and acorn squashes, with the vegetable group (19). The nutrient compositions of white and sweet potatoes are quite similar. Sweet potatoes do have more beta-carotene (20) but white potatoes have far more potassium (9).

Given these inconsistencies, a tool that addresses the quality of carbohydrate-containing foods would be a meaningful addition to inform the categorization of foods for development of food-based dietary guidelines.

## Carbohydrate quality: an important consideration for dietary recommendations

At a time when most Americans do not eat enough vegetables, moving a group of vegetables to a different food group, could result in vegetable consumption in food assistance programs and other efforts affected by DGA. Given the diverse variety and nutrient composition of carbohydrate-containing foods, others have suggested the usefulness of an evidence-based metric for measuring carbohydrate food quality (21). It has been postulated that such a tool would be useful for informing not only nutrition researchers, but also food developers and those setting future public dietary guidance, including the DGAC. When multiple nutrient profiling methods were applied to high-carbohydrate foods, the authors concluded that the nutrient content of white potatoes warranted greater priority in dietary recommendations (22).

Among these justifications for a more holistic metric of carbohydrate quality was better alignment with current dietary guidance, including a focus on micronutrients to encourage, as well as ones to limit. Current global dietary guidelines encourage consumption of whole grains and many, including the 2020–2025 DGA, note fiber and potassium among the “nutrients of concern” because more than half the population consumes insufficient amounts. Additionally, the DGAs have consistently encouraged reduction of saturated fat, sodium, and added sugars, due to historical overconsumption by most consumers.

The result was the development of a metric known as the Carbohydrate Food Quality Score (CFQS), which builds onto the 10:1:1 model of carbohydrate:fiber:free sugar first developed by Liu et al., and considers sodium, potassium, added sugars, and whole grains, all of which were addressed in the 2020–2025 DGAs (23). More research using this score to address the health outcomes of consuming whole grains, and various dietary patterns is warranted. Thus far, the CFQS correlates strongly with two other measures: the Nutrient-Rich Food Index and the Nutri-Score, with a more robust assessment of carbohydrate quality.

## Menu modeling analysis demonstrates nutrition consequences of swapping starchy vegetables and grains

The question regarding interchangeability of starches and grains suggests a need to evaluate their unique nutrient contributions. A comparison of the nutrients these foods provide through menu modeling is one way to understand the differences between starchy vegetables and grains and demonstrate how they are not nutritionally similar or interchangeable.

Table 1 shows a 2,000 kcal “foundation” menu that aligns with USDA’s “Food Pattern Models” and the 2020–2025 DGA (24).

**Table 1:** Menu modeling comparison.

	Menu #1—Foundational menu—healthy United States (2,092 kcal)	Menu #2—Replace 100% starchy vegetables with grains (2021 kcal)
Breakfast	Scrambled eggs: Two eggs Cooked in 0.5 TBSP olive oil	Scrambled eggs: Two eggs Cooked in 0.5 TBSP olive oil
	Hash browns: 2 oz. hash browns	Toast: 2 oz whole wheat bread
	Toast: 1 oz. whole wheat bread	Orange: 1/2 medium orange
	Orange: 1/2 medium orange	Coffee: 8 oz coffee
	Coffee: 8 oz. coffee	
Morning snack	Yogurt with corn flakes, fruit: 1/2 cup nonfat Greek yogurt 1/2 cup corn flakes 1/4 cup blueberries	Yogurt with corn flakes and fruit: 1/2 cup nonfat Greek yogurt 1/2 cup corn flakes 1/4 cup blueberries
Lunch	Spinach/chicken salad and whole wheat pita with a side of blue corn chips: One cup spinach 1/2 cup whole cherry tomatoes One TBSP Ranch dressing 2 oz. roasted skinless chicken breast 0.25 oz. sunflower seeds One large whole wheat pita pocket 1 oz. blue corn tortilla chips	Spinach/chicken salad and whole wheat pita with a side of blue corn chips: One cup spinach 1/2 cup whole cherry tomatoes One TBSP Ranch dressing 2 oz. roasted skinless chicken breast 0.25 oz. sunflower seeds One large whole wheat pita pocket 1 oz. blue corn tortilla chips
	Milk: 8 oz. nonfat milk fortified w/vitamins A & D	Milk: 8 oz. nonfat milk fortified w/vitamins A & D
	Apple: One medium apple	Apple: One medium apple
Afternoon snack	Cheese and popcorn: 0.75 oz. cheddar cheese, reduced fat Three cups air popped popcorn	Cheese and popcorn: 0.75 oz. cheddar cheese, reduced fat Three cups air popped popcorn
Dinner	Steak, baked potato, and broccoli, and chocolate: 3 oz. broiled lean top sirloin 5 oz. baked russet potato, skin on (medium potato) 1 tsp. sour cream 1/2 cup boiled broccoli	Steak, rice, and broccoli: 3 oz. broiled lean top sirloin *½ cup white rice, cooked* *One pat butter, unsalted* 1/2 cup boiled broccoli
	Milk: 8 oz. nonfat milk fortified w/ Vitamins A & D 1 oz. dark chocolate	Milk: 8 oz. nonfat milk fortified w/ Vitamins A & D 1 oz. dark chocolate
[Menu footnote]	This menu item follows the Healthy United States dietary pattern and features a variety of whole grain and refined grains	Note on page 2 of the food pattern reports for Healthy United States-Style Eating Patterns that vitamins D and E is usually low (46 and 68%, respectively for adult males and females). The foundation menu meets or exceeds the % RDA reflected in this perspective

Table 1 also outlines a comparison menu, and is also aligned with 2020–2025 DGAs, but this menu replaces the starchy vegetables in Menu 1 with grains, with at least half the grains being whole grains. Both meal patterns provide approximately 2,000 cal. These findings (detailed in Table 2) highlight the nutritional differences between potatoes and grain foods and underscore the importance of maintaining potatoes in the vegetable group. While swapping grains for potatoes may increase intakes of some key nutrients, others will decrease significantly. Consuming grains instead of potatoes (the no starchy vegetable diet) may result in several nutritional risks including:

- Decreased intake of potassium (−21%).
- Shortfalls in dietary fiber intake (−10%).
- Reduced intake of choline (−2%) and vitamins B6 (−17%), C (−11%), and E (−5%).

**Table 2:** Nutrient differences when starchy vegetables are replaced with grains.

Nutrient	% Change − 100% Starchy vegetable replaced with grains (i.e., 100% grains) menu
Potassium	**−21%**
Sodium	**−12%**
Calcium	+1%
Choline	−2%
Dietary Fiber	**−10%**
Vitamin D	0%
Vitamin B1	+7%
Vitamin B6	**−17%**
Vitamin B12	0%
Vitamin C	**−11%**
Vitamin E	−5%
Folate	+5%
Iron	+1%
Zinc	+1%

Bold values are nutrient variances of at least 10%.

Of particular concern are fiber and potassium, considered as “nutrients of public health concern” by the 2020–2025 DGA (page 49), because “low intakes are associated with health concerns.” Replacing potatoes with grains resulted in a considerable decrease in potassium content (−21%). Many starchy vegetables are good sources of potassium (e.g., one medium potato provides 15% DV). Consuming grains in place of starchy vegetables may further widen the gap between recommended and actual intakes of potassium. A meaningful decrease in fiber intake (−10%) also occurred when grains replaced potatoes. This finding reinforces the need to include both starchy vegetables and grains in the diet to obtain adequate intake of fiber, especially given that currently 90% of women and 97% of men do not meet dietary fiber intake recommendations (25). It is important to emphasize that, while potatoes are a vegetable, they are not a replacement for, nor interchangeable with, non-starchy vegetables. Currently, only one in 10 adults meets recommendations for vegetables so consumption of all varieties of vegetables should be encouraged (26).

## Discussion

To minimize confusion about carbohydrate recommendations, it is critical to clarify the unique differences between starchy vegetables and grains given their significant nutrient contributions to the American diet. Nutrition analysis clearly demonstrates that starchy vegetables, including potatoes, are not nutritionally interchangeable with grains. Both are high in carbohydrates, but their micronutrient contributions are different and deserve dietary recommendations that respects this difference.

As with grains, a diverse intake of starchy vegetables should be encouraged. The limitation of these menu modeling results is that potatoes were the only starchy vegetable included. Additional modeling done with other culturally relevant starchy vegetables, and over 7 days or more, may show different results.

This perspective addresses white potatoes, in part because they are the most commonly consumed starchy vegetable and have the same nutritional strengths as other starchy vegetables, particularly with respect to their content of potassium, vitamin C, and dietary fiber. Those with color in the edible portion, such as butternut and acorn squash, also contain beta-carotene, as do sweet potatoes, but other starchy vegetables do not, such as beets and cassava (yuca). Yet all, except white potatoes, are grouped as vegetables in much of the scientific literature that uses the aforementioned FFQ.

Carbohydrate guidance should focus not only on the unique nutritional assets of each, but also their varied culinary uses and cultural relevance within diverse foodways. All starchy vegetables have nutrient profiles that are distinct from those of grain foods and should retain their categorizations as separate food groups in dietary guidance.

## Data availability statement

The original contributions presented in the study are included in the article/supplementary material, further inquiries can be directed to the corresponding author.

## Author contributions

KA: Writing – original draft, Writing – review & editing.

## Funding

The author(s) declare financial support was received for the research, authorship, and/or publication of this article. Funding for this perspective was provided by Potatoes USA.

## Conflict of interest

This study received funding from Potatoes USA. The funder had the following involvement with the study: composition and data analysis of the menu modeling. KA was compensated by Potatoes USA for preparation and revision of the manuscript.

The author(s) declared that they were an editorial board member of Frontiers, at the time of submission. This had no impact on the peer review process and the final decision.

## Publisher’s note

All claims expressed in this article are solely those of the authors and do not necessarily represent those of their affiliated organizations, or those of the publisher, the editors and the reviewers. Any product that may be evaluated in this article, or claim that may be made by its manufacturer, is not guaranteed or endorsed by the publisher.
